# Choice of population database for forensic DNA profile analysis

**DOI:** 10.1016/j.scijus.2014.10.004

**Published:** 2014-12

**Authors:** Christopher D. Steele, David J. Balding

**Affiliations:** UCL Genetics Institute, Darwin Building, Gower Street, London WC1E 6BT, UK

**Keywords:** WoE, Weight of Evidence, Q, Queried contributor, X, Alternate contributor that replaces Q in defence hypothesis, K, Contributor to the CSP whose reference profile is available, U, Unprofiled contributor to the CSP, Population database, DNA mixtures, Likelihood ratio, Forensic DNA

## Abstract

When evaluating the weight of evidence (WoE) for an individual to be a contributor to a DNA sample, an allele frequency database is required. The allele frequencies are needed to inform about genotype probabilities for unknown contributors of DNA to the sample. Typically databases are available from several populations, and a common practice is to evaluate the WoE using each available database for each unknown contributor. Often the most conservative WoE (most favourable to the defence) is the one reported to the court. However the number of human populations that could be considered is essentially unlimited and the number of contributors to a sample can be large, making it impractical to perform every possible WoE calculation, particularly for complex crime scene profiles. We propose instead the use of only the database that best matches the ancestry of the queried contributor, together with a substantial *F*_*ST*_ adjustment. To investigate the degree of conservativeness of this approach, we performed extensive simulations of one- and two-contributor crime scene profiles, in the latter case with, and without, the profile of the second contributor available for the analysis. The genotypes were simulated using five population databases, which were also available for the analysis, and evaluations of WoE using our heuristic rule were compared with several alternative calculations using different databases. Using *F*_*ST*_ = 0.03, we found that our heuristic gave WoE more favourable to the defence than alternative calculations in well over 99% of the comparisons we considered; on average the difference in WoE was just under 0.2 bans (orders of magnitude) per locus. The degree of conservativeness of the heuristic rule can be adjusted through the *F*_*ST*_ value. We propose the use of this heuristic for DNA profile WoE calculations, due to its ease of implementation, and efficient use of the evidence while allowing a flexible degree of conservativeness.

## Highlights

• A heuristic rule of assuming the database of Q for all unprofiled individuals in a CSP is proposed.
• We simulate a total of 105 000 one- and two-contributor CSPs with no dropin or dropout.
• The heuristic rule is conservative compared to an alternative for the majority of simulated CSPs.
• We suggest that the use of this heuristic will allow for evaluation of complex cases with many possible databases.

## 1 Introduction

In forensic DNA analysis, unknown contributors to a DNA profile are usually considered to come from one of several populations for which an allele frequency database is available. The choice of database can have an important impact on weight of evidence (WoE): the rarer an allele the stronger the evidence implicating a queried contributor (Q) if he has that allele and it is observed in the crime scene profile (CSP). The most appropriate population is the one that best matches the ancestry of X, the true source of the DNA. Under the prosecution case X is assumed to be Q, but under the defence case there is often little or no information about the ancestry of X. Many authors have noted that the database most appropriate for Q is not necessarily most appropriate for X [6,4]. Conversely, [3] argue for using the database of Q even if the ancestry of X is unknown, in part because the observation of the profile of Q introduces a size-bias effect: an observed profile tends to be more common in the population in which it was observed than in a different population. Thus, having observed the profile of Q, on average the probability for X to have the same profile is higher if X is assumed to come from the same population.

In current forensic practice, when the ancestry of X is unknown, it is common to consider multiple population databases and choose the one that generates the lowest WoE. There should be no requirement to favour defendants in this way. Suppose for example that Q is Caucasian but it is discovered that the lowest WoE is obtained using a database of Vietnamese individuals. If the population local to the crime includes few Vietnamese and there is no evidence to suggest that a Vietnamese person was the source of the DNA, it may not be helpful to the court to report the WoE arising from the Vietnamese database. Similarly, the world's population can be categorised in a vast number of different ways, and it is not possible to investigate them all in order to report the smallest WoE. However, a forensic expert should make reasonable allowance for the different possible ancestries of X, given the available knowledge about the location and nature of the crime. It can be expedient to make approximations that favour the defence in order to permit simplified analyses while avoiding courtroom challenges. Here we propose a heuristic for WoE analysis that involves only one calculation, using the database most appropriate for Q. We show that our heuristic tends to strongly favour defences compared with a range of alternative calculations.

For a one-contributor CSP when there are only, say, five population databases, it is usually easy to compute the WoE for each database and choose the one most favourable to the defence. However, for mixed profiles, the computational effort to consider multiple databases for each unprofiled contributor can be substantial. Thus our heuristic that computes the WoE only using the database of Q would be attractive, provided that it can be established to be conservative (favourable to the defence). If X is from the same population as Q then it becomes relevant to consider that they may also come from the same subpopulation, in which case an *F*_*ST*_ adjustment may be required [3]. We have recently published worldwide *F*_*ST*_ estimates appropriate for forensic use [7] and concluded that choosing *F*_*ST*_ = 0.03 is sufficiently large to be almost always conservative. The effect of the *F*_*ST*_ adjustment is to increase the probability assigned to the alleles of Q, and consequently decrease the probability for other alleles. Although the rationale for an *F*_*ST*_ adjustment is to allow for the possibility that X has ancestry similar to that of Q, we illustrate below that for *F*_*ST*_ = 0.03 our heuristic calculation is conservative even if X could have come from one of several different populations. It is for this reason that our heuristic uses the same value of *F*_*ST*_ whatever the population of Q, even though within-population *F*_*ST*_ values differ across populations.

A similar argument applies to other contributors to a mixed CSP. Consider a two-contributor profile, one of the contributors being X, who is alleged to be Q. If the reference profile of the other contributor is known, as is often the case for a victim or bystander, there are no probabilities to assess for the alleles of that individual and so the question of the appropriate population database is essentially the same as for the one-contributor case. When the reference profile of the other contributor, say U, is unavailable, then we show that it is conservative to use for both X and U the database best matching the ancestry of Q, again with *F*_*ST*_ = 0.03. The *F*_*ST*_ adjustment under our heuristic only increases the population allele fraction for the alleles of Q, which is helpful to defences because it increases the probability that X or U share alleles with Q, thus increasing the support for the defence explanation of the observed CSP.

It is not feasible or desirable to guarantee that a proposed WoE calculation is more favourable to the defence than any conceivable alternative calculation. We perform simulation experiments which show that for UK population databases our heuristic WoE calculation is, with probability ≫ 0.99, more favourable to defendants than a range of reasonable alternative calculations. We first simulate single-contributor CSPs matching the reference profile of the alleged contributor Q. Then the WoE for Q to be a contributor is calculated using the correct database (that used for the simulation) and is compared with the smallest WoE calculated using in turn four other databases. We repeated this exercise for one database using allele fractions that differ from the database values according to each of three values of *F*_*ST*_, and show that our heuristic remains conservative compared to the WoE from the four alternative databases.

We then simulate two-contributor CSPs using all possible choices of two databases from the five available, and compare the WoE computed using the database of Q for both contributors (and *F*_*ST*_ = 0.03) with (a) the correct assignment of databases, (b) the minimum WoE using each of the four alternative databases for both X and U, and (c) the minimum WoE over the four databases for X, always using the correct database for U. In all our calculations, an adjustment using *F*_*ST*_ = 0.03 is applied to the alleles of Q when the database of Q is used for X.

When a calculation is performed using a database different from that of Q, perhaps because of evidence about the ethnic background of X, coancestry is not relevant and so it is appropriate to use *F*_*ST*_ = 0. It has been suggested [2] that even in this setting it would be cautious to use a low value of *F*_*ST*_ such as 0.01. This introduces some bias in favour of the defendant in order to allow for the ancestry of X to differ somewhat from the database population. Here we assume that there is no specific suggestion of an alternative population for X, and since a bias in favour of defendants is introduced by taking the minimum WoE over four alternative database choices, we use *F*_*ST*_ = 0 in calculations using databases different from that of Q.

It is possible that the true ancestry of Q is unknown or misassigned, for example if he impersonates another individual, or an assessment of his physical appearance was incorrect. He may also be of mixed ancestry or some other ancestry not well represented in the available databases. In that case there is no size-bias effect tending to make the observed profile of Q more common in the population to which he is assigned than in other populations. However, although such an error may have an adverse impact on the calculated WoE, the generous value of *F*_*ST*_ is the main factor underlying the conservative nature of the WoE analysis that we propose, and so the impact of any population misassignment of Q will be relatively small.

## 2 Materials & methods

### 2.1 Databases

We have used frequency data at 16 STR loci for five UK populations: Caucasian (IC1), African and African Caribbean (IC3), South Asian (IC4), East Asian (IC5) and Middle Eastern (IC6) (Table 1). For further details of the dataset, see [7]. We used these data to simulate 16-locus profiles assuming Hardy–Weinberg and linkage equilibria. Neither dropin nor dropout are included in the simulations, nor are they allowed for in the analyses.

The WoE is computed using the likelihood ratio framework [5], and reported in bans (= *log*_10_(likelihood ratio)) comparing a hypothesis that includes Q as a contributor with an alternative in which Q is replaced by X, assumed to be unrelated to Q. We implement *F*_*ST*_ adjustment [2] to the population fractions of the alleles of Q whenever the database most appropriate for Q is used for X; the adjustment uses *F*_*ST*_ = 0.03, and otherwise *F*_*ST*_ = 0. In all calculations one was added to the database count for each allele of Q, introducing a bias against understating the frequencies of rare alleles [1].

### 2.2 Simulation experiments

Initially a series of 10 000 one-contributor CSPs were simulated, using in turn allele fractions from each of the five population databases (so 50 000 profiles in total). The WoE for each simulated CSP was calculated five times, each time comparing hypotheses of the form:

$$\begin{array}{l} H_{p}:\;Q\\ H_{d}:\;X \end{array}$$

but using a different database. The minimum WoE over the four incorrect databases was then subtracted from the WoE computed using our heuristic (which uses the database of Q and *F*_*ST*_ = 0.03), so that a negative result indicates that it is favourable to the defence to report our heuristic WoE irrespective of the ancestry of X.

A second set of one-contributor analyses was conducted to investigate the effect of Q having an ancestry that differs from all of the available databases. Simulations were based on the IC1 database but with allele fractions differing from the IC1 values according to three *F*_*ST*_ values (0.01, 0.02, and 0.03). Ten thousand CSPs were simulated for each *F*_*ST*_ value (30 000 in total). The hypotheses compared were the same as above, and our heuristic was again applied (using the IC1 database) from which was subtracted the minimum WoE using each of the four other databases.

Next, 25 sets of 1000 two-contributor profiles were created, one for each choice of databases for the two contributors. The hypotheses compared were of the form:

$$\begin{array}{l} H_{p}:\;Q+K\\ H_{d}:\;X+K \end{array}$$

where K denotes that the second contributor was known (the reference profile was available for the analysis). The WoE computed using our heuristic was compared with the minimum WoE computed using each of the four alternative databases.

We then performed a series of analyses based on the same simulations but now assuming that the uncontested contributor to the two-contributor profiles was unknown, and so the hypotheses compared were of the form:

$$\begin{array}{l} H_{p}:\;Q+U\\ H_{d}:\;X+U \end{array}$$

For each dataset we computed the WoE using our heuristic with three alternative WoE calculations.

The first alternative WoE calculation used the correct database for each of X and U, which differs from our heuristic in the 20 datasets with Q and U simulated from different databases. This alternative may be regarded as the most appropriate WoE, while our heuristic WoE is biased in favour of the defence because the *F*_*ST*_ adjustment increases the probability for U to share alleles with Q. The second alternative, applicable in all 25 datasets, uses the lowest WoE obtained over all possible databases for X, using the correct database for U. The third alternative WoE calculation, also applied in all 25 datasets, uses the lowest WoE over the four alternative databases, the same for both X and U.

## 3 Results

Table 2 shows summary results for the five simulation experiments. As the difficulty of the inference problem increases and the mean WoE decreases, the mean difference between our heuristic and the alternatives considered also decreases in absolute terms, but increases as a percentage of the alternative WoE. We have not considered three or more contributors because the computational demands of a large simulation study are prohibitive, but this trend suggests that for CSPs with three or more contributors, the mean difference between our heuristic and the alternative WoE would be a large percentage of the latter.

**Fig. 1:**
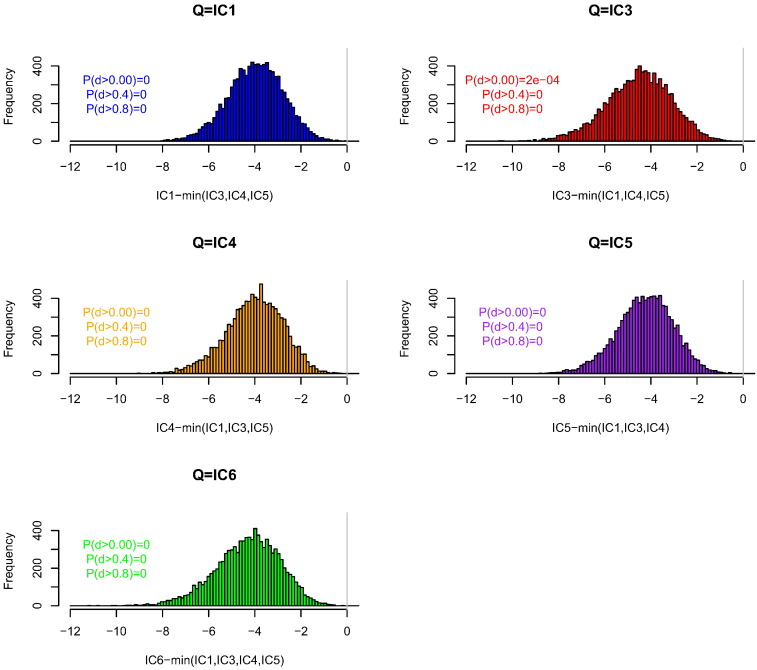
The effect of database on weight of evidence (WoE) calculations for a one-contributor CSP. The databases are described in Table 1. The x-axis shows the WoE computed using the database from which the contributor Q was simulated (indicated in the subplot title) with *F*_*ST*_ = 0.03, minus the lowest WoE computed using each of the four alternative databases and *F*_*ST*_ = 0. P(d > x) indicates the proportion of differences that are > x.

In one-contributor tests, our heuristic gives, with probability > 0.999, a lower WoE than any of the four alternative calculations (Fig. 1). There were two instances in 50 000 simulated profiles of an advantage to the defence from using one of the alternative databases. On average, the WoE obtained using our proposed calculation is lower by 0.3 bans (1 ban = 1 order of magnitude) per locus than the minimum over the four alternative calculations (Table 2, column 1). When the tested individuals are not simulated directly from the database allele frequencies, but differ according to *F*_*ST*_ = 0.01, 0.02 and 0.03, we found that the number of comparisons that are not conservative is at most 3 out of 10 000, which is as expected higher than when Q is simulated directly from the IC1 database (0 non-conservative out of 10 000) but the difference is small and not significant.

Including a known contributor reduces the WoE for both our heuristic and the minimum of the four alternatives by about 3 bans (Table 2, column 2). The difference between them remains similar to that for the one-contributor analyses (column 1). The fraction of simulations in which our heuristic was conservative ranged from 0.994 to 0.999 across the five databases used to simulate Q.

**Fig. 2:**
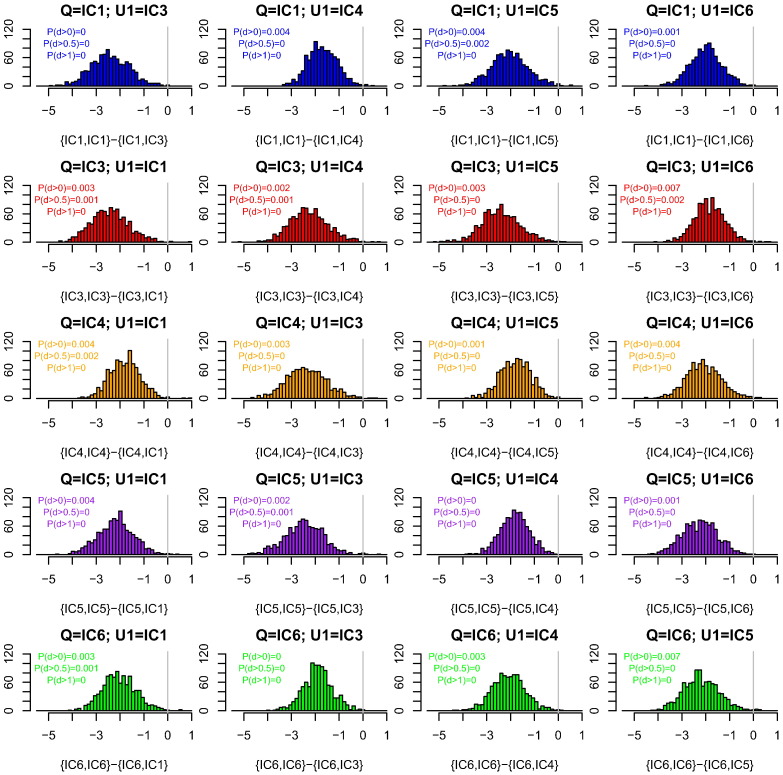
The effect of database on weight of evidence (WoE) for two-contributor CSPs. The databases are described in Table 1. The x-axis shows the WoE computed using the database of Q for both contributors minus that obtained using the correct databases for X and U. The title of each subplot indicates the databases from which each contributor was simulated, where Q is the queried contributor and U is an unknown contributor. The x-axis labels indicate the databases used for each contributor in the analysis. P(d > x) indicates the proportion of differences that are > x. Colour indicates the database of Q.

When the additional contributor is unknown (U rather than K), the fraction of simulations in which our heuristic was conservative compared with using the correct databases for each of X and U was on average 0.997, and at least 0.993 over the 20 choices of databases for X and U (Fig. 2). The reason that our heuristic is conservative is that it is helpful to the defence to maximise the probability that U has alleles matching those of Q, and this is achieved in our heuristic using the database of Q together with *F*_*ST*_ = 0.03. The probabilities assigned to alleles of U not shared with Q are less important because these have a similar effect under both prosecution and defence hypotheses. Using our heuristic, P(WoE > 9) = 0.903, and so the LR is usually but not always in excess of one billion.

**Fig. 3:**
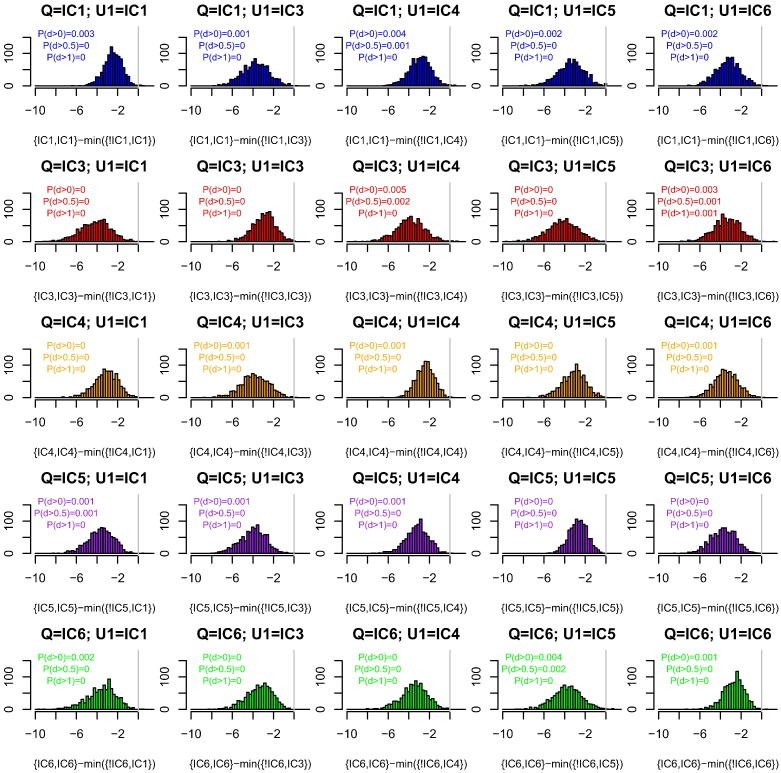
The effect of database on weight of evidence (WoE) for two-contributor CSPs. The databases are described in Table 1. The x-axis shows the WoE computed using the database of Q for both contributors minus the minimum WoE obtained over all other choices of databases for X, always using the correct database for U. The title of each subplot indicates the databases from which each contributor was simulated. The x-axis labels indicate the databases used for each contributor in the analysis (!IC1 indicates all databases other than IC1). P(d > x) indicates the proportion of differences that are > x. Colour indicates the database of Q.

**Fig. 4:**
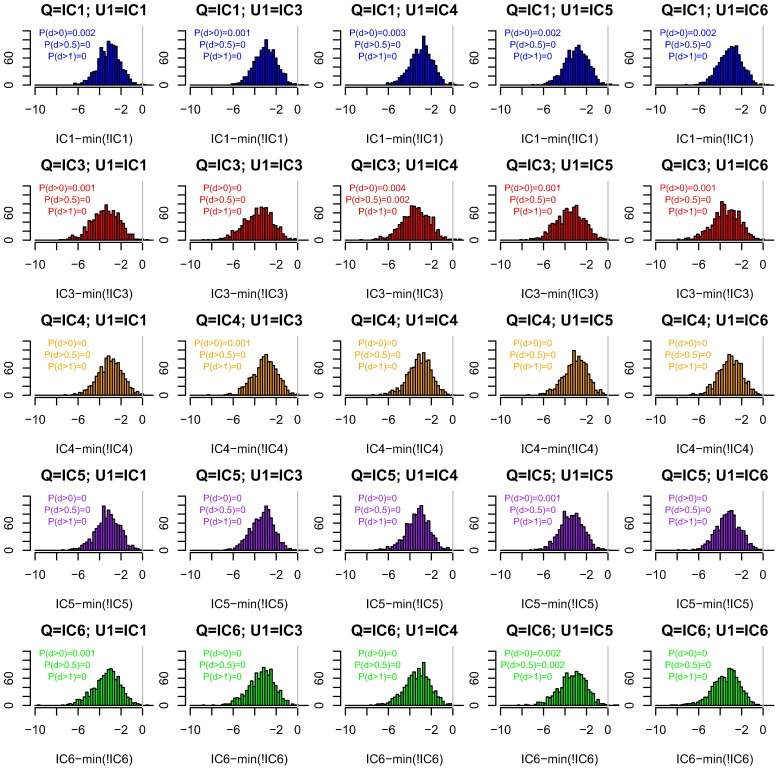
The effect of database on weight of evidence (WoE) for two-contributor CSPs. The databases are described in Table 1. The x-axis shows the WoE computed using the database of Q for both contributors minus the minimum WoE obtained over using each other database in turn for both X and U. The title of each subplot indicates the databases from which each contributor was simulated. The x-axis labels indicate the database used for both contributors in the analysis. P(d > x) indicates the proportion of differences that are > x. Colour indicates the ancestry of Q.

Fig. 3 shows that the WoE computed under our heuristic is almost always (*P* > 0.995) less than the minimum value over the four alternative choices of database for X, with U always assigned the correct database. Finally, Fig. 4 shows that if the same database is used for both X and U, it is conservative (*P* > 0.996) to use our heuristic.

## 4 Discussion

We have shown that for a one-contributor setting, our heuristic WoE calculation that uses only the database of the queried contributor Q is almost always conservative (favours defences) compared with choosing the lowest WoE among four other databases for the alternative contributor X (Fig. 1). Similar results hold when there is additionally an uncontested contributor K with reference profile available. When the additional contributor is unprofiled, using the database of Q for both X and U is almost always conservative compared to (a) using the correct database for each of X and U (Fig. 2), (b) using the correct database for U, and choosing the most favourable alternative database for X (Fig. 3), and (c) choosing the most favourable among alternative databases, that database being used for both X and U (Fig. 4). In all calculations we used *F*_*ST*_ = 0.03 when the database of Q was used, and *F*_*ST*_ = 0 otherwise. The *F*_*ST*_ adjustment increases the population probabilities for the alleles of Q, but not other alleles observed in the CSP nor any other available reference profiles.

In all our simulations, our heuristic is conservative compared with the alternative calculations considered in at least 99.3% of the simulations, and in the few instances that it was not conservative the difference was always < 1.5 bans. The world's population can be divided into an unlimited number of different subpopulations; therefore there can be no precisely correct choice of alternative subpopulations to consider. What is required is an average WoE over each possibility for the ancestry of the alternative contributor X, weighted by its plausibility given the known circumstances of the crime. Our heuristic will almost certainly give a result that is more favourable to defendants. We have verified that our good results are not favourably biased because Q is sampled from the same database used in the analysis.

The degree to which our heuristic favours defences can be controlled by changing the value of *F*_*ST*_ from 0.03 used here. [7] found that *F*_*ST*_ = 0.03 exceeds almost all median *F*_*ST*_ values from world-wide comparisons of subpopulations with continental-scale populations, and we have shown here that it also suffices to ensure that using the database of Q with this *F*_*ST*_ value almost always returns a lower WoE than a range of alternative calculations.

We have not performed simulations for three or more unknown contributors because of the prohibitive time required for a simulation study, but the same principles apply to ensure that our heuristic will be similarly conservative. The large average reduction in WoE compared with the two-contributor case suggests that the difference in WoE will also be reduced, although it is expected to increase as a fraction of the overall WoE (see Table 2).

We believe that our heuristic offers a good policy for WoE calculation based on DNA evidence that is easy to implement, and almost always favourable to defendants relative to reasonable alternative policies. Because it is favourable to defendants to use the database most appropriate for Q, it will therefore generally be unfavourable to defendants if the wrong database is used because the ancestry of Q is misassigned, or because there is no appropriate database. However the relatively large value of F_ST_ is the main factor in ensuring that our heuristic tends strongly to favour defendants, and so while misassigning the database of Q will have some detrimental impact, it will usually be small and outweighed by the impact of the *F*_*ST*_ value.

## Figures and Tables

**Table 1:** Number of allele observations at each locus for each population database: Caucasian (IC1), Afro-Caribbean (IC3), South Asian (IC4), East Asian (IC5) and Middle Eastern (IC6).

Allele counts	IC1	IC3	IC4	IC5	IC6
D3S1358	6878	3941	520	599	1202
TH01	6816	3918	514	598	1202
D21S11	6870	3941	520	599	1199
D18S51	6808	3930	520	600	1195
D16S539	6818	3927	514	600	1199
VWA	6877	3936	520	600	1201
D8S1179	6871	3941	520	600	1202
FGA	6853	3938	516	600	1201
D19S433	6702	3868	507	595	1197
D2S1338	6443	3758	491	594	1176
D22S1045	1816	2482	421	498	954
D1S1656	1827	2508	426	504	959
D10S1248	1815	2499	416	500	912
D2S441	1800	2473	420	493	943
D12S391	1857	2543	437	499	945
SE33	368	872	237	394	268

**Table 2:** Mean weight of evidence (WoE) for the heuristic rule and the alternatives discussed in the text. The mean of the differences between the heuristic and alternative scenarios is also shown. The % Difference row shows the mean difference as a percentage of the average of the heuristic and alternative means.

Contributors under Hd	X	X + K		X + U	
			True both	True U	Same dbase
Heuristic (bans)	20.3	17.8	10.7	10.7	10.7
Alternative (bans)	24.5	20.7	12.8	14.1	14.0
Difference (bans)	4.2	3.0	2.1	3.4	3.2
Difference (%)	18.8	15.6	17.9	27.4	25.9
